# Between being healthy and becoming comatose: the neuropsychiatric landscape of critical illness with a focus on delirium, DSM-5 and ICD-11

**DOI:** 10.1186/s12888-019-2201-9

**Published:** 2019-07-16

**Authors:** Jan N. M. Schieveld, Emma H. C. W. van de Riet, Jacqueline J. M. H. Strik

**Affiliations:** 1 Department of Psychiatry and Psychology, Division of Child and Adolescent Psychiatry and Psychology, Maastricht University Medical Center, P.O. Box 5800, 6202 AZ Maastricht, The Netherlands; 2 School for Mental Health & Neuroscience (MHeNS), University of Maastricht, Maastricht, The Netherlands

**Keywords:** Neuropsychiatry, Delirium, Critical illness, ICD-11, DSM-5

## Abstract

**Background:**

One of the most important questions remaining in matters of critical illness in the year 2019 is arguably how to address the diverse neuropsychiatric complications of critical illness.

**Main text:**

The ICD-11 and DSM-5, two of the world’s leading classification systems, disagree regarding important aspects of delirium; moreover, they do not mention critical illness and its neuropsychiatric complications at all.

**Conclusions:**

It would have been desirable for the committees revising the DSM-IV-TR and ICD-10 to have joined forces in order to generate classification systems that complement each other and, moreover, that address the “The Neuro-Psychiatry of Critical Illness”.

## Background

The number of intensive care unit (ICU) admissions annually due to critical illness, and the associated mechanical ventilation episodes and days, is one of the fastest growing numbers in clinical medicine in the western world. Critical illness can cause the brain to react in the form of disruptive neuropsychiatric symptoms and disorders, the most well-known being delirium. As early as 1980, ZJ Lipowski stated: “Delirium is acute brain failure in man” [1]. This awareness is important, as a healthy brain, which also directs the autonomic nervous system and endocrine systems, is crucial in surviving critical illness.

## Main text

The ten most common neuropsychiatric reaction types of the brain are: fever, sickness behavior, executive function problems, epilepsy, hyperactive delirium, acute apathy syndrome (a more appropriate name than hypoactive delirium), catatonic agitation or inhibition, refractory agitation or inhibition, coma, and death [2]. These are all a result of the critical illness itself as well as the psychotropic medication administered.

Sickness behavior is characterized by fatigue, irritability or emotional lability, loss of initiative, anorexia, and isolation. This is a very common reaction pattern and it is particularly related to infections, cancer, and chemotherapy. Executive function problems are problems with attention, short-term memory, planning, learning, and organizing.

The abovementioned neuropsychiatric symptoms and disorders are accompanied by disturbances of consciousness (reflected in Richmond Agitation-Sedation Scale (RASS) score), frequently along with agitation or inhibition syndromes [1, 3]. They can be accompanied by acute psychotic features, especially visual and acoustic hallucinations, and paranoid delusions.

The neuropsychiatric complications of critical illness are not only closely interrelated, but also directly and indirectly related to worse patient outcomes on the short run (like length of hospital stay, morbidity, and mortality) and in the long run the final level of psycho-socio-emotional functioning. Unfortunately, many survivors of critical illness suffer from brain injury (non-traumatic), post-traumatic stress disorder (PTSD), anxiety, or depression, as do many of their family members [4]. Lastly, the burden of these symptoms also involves huge medical and non-medical costs.

So how can we make sense of this very complicated yet clinically highly important variable and fluctuating neuropsychiatric landscape? And how can we get a quick and easy overview of all these neuropsychiatric reaction types of the brain at the patient’ bedside?

One step forward is the introduction of the ABCDEF bundle [5]. This is an evidence-based guide for all medical and paramedical specialists and nursing teams alike to develop and approach the multidisciplinary organizational changes required for optimizing ICU-patient recovery and outcomes. The ABCDEF bundle includes: Assess, prevent, and manage pain; organize both spontaneous awakening and spontaneous Breathing and organize multidisciplinary Brain rounds; organize well-considered Choice of analgesia and sedation; assess, prevent, and manage Delirium; assess and manage Early mobility and Exercise; and organize Family empowerment and engagement [5].

However, neither the fifth edition of the diagnostic and statistical manual of mental disorders (DSM-5) nor the recently published and yet to be adopted eleventh revision of the international classification of diseases (ICD-11), the number one classification system in the world, hardly mention or do not mention at all neuropsychiatric symptoms and disorders in the critically ill [6, 7]. There are discrepancies between DSM-5 and the ICD-11regarding delirium, especially regarding the existence of the three delirium motor types: hyper-“hypo”(acute apathy syndrome) and mixed. This clinically highly important distinction is made in the DSM-5 but not in the ICD-11. Moreover, the DSM-5 and the ICD 11 share the same major flaw: they appear to disregard the existence of the concept of critical illness entirely, and this is truely concerning. The DSM-5 only mentions the existence of “intensive care” once, on page 600, and this is in relation to the very high prevalence of delirium there, while the ICD-11 does not contain the search terms “critical illness” at all.

**Fig. 1:**
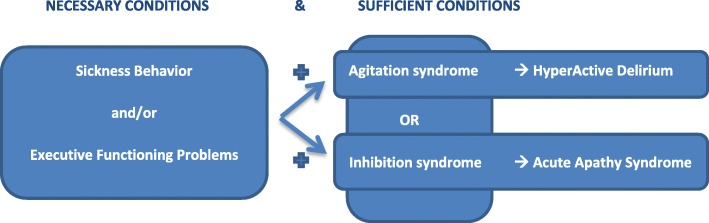
Two final, and different, neuropsychiatric syndromes in critical illness

Specifically, they do not discuss sickness behavior, executive function problems, or acute apathy syndrome, nor do they agree about some major issues relating to delirium such as: 1) which criteria are *necessary?* (e.g. illness/critical illness, level of consciousness, sickness behavior, and/or executive function problems); 2) which criteria are *sufficient* (plus an agitation and/or inhibition syndrome)?; 3) which subtypes of delirium exist? and 4) which are the main type-specifying criteria? [8] (Fig. 1).

Addressing these questions is of ever-growing clinical relevance [8]. This is also illustrated by a very recent paper in which the authors treated “delirium with antipsychotics” [9]. However, as they did specify the delirium type, they next did not specify the unique targeted symptoms by the antipsychotics [1, 10]. Specifying the delirium type is important, as acute apathy syndrome differs from hyperactive delirium in four major aspects: its symptoms, its prevalence (higher), the treatment response (lower/none), and the outcome (worse) [2].

## Conclusions

It would have been logical for the committees revising the DSM-IVTR and ICD-10 to have joined forces in order to generate classification systems that complement each other and, moreover, that address the aforementioned issues in a section entitled “The Neuro-Psychiatry of Critical Illness”. This section will be highly relevant for medical and non-medical specialists, patients, their families, and other parties. It will direct future scientific research, enhance awareness and understanding of neuropsychiatric symptoms and disorders in the critically ill, and thus improve patient and family care, outcomes, and well-being.

## Data Availability

Not applicable.

## Abbreviations

DSM-5: fifth edition of the diagnostic and statistical manual of mental disorders
ICD-11: eleventh revision of the international classification of diseases
ICU: Intensive care unit
PTSD: Post-traumatic stress disorder
RASS: Richmond Agitation-Sedation Scale
